# BrainFilm, a novel technique for physical compression of 3D brain slices for efficient image acquisition and post-processing

**DOI:** 10.1038/s41598-018-26776-9

**Published:** 2018-06-04

**Authors:** Joo Yeon Kim, Hyun Jung Kim, Min Jee Jang, June Hoan Kim, Ju-Hyun Lee, Eunsoo Lee, Kyerl Park, Hyuncheol Kim, Jaedong Lee, Jeehyun Kwag, Namhee Kim, Mi-Ryoung Song, Hyun Kim, Woong Sun

**Affiliations:** 1 Department of Anatomy and Division of Brain Korea 21 Plus Biomedical Science, College of Medicine, Korea University, Seoul, 02841 Korea; 2 Department of Brain and Cognitive Engineering, Korea University, Seoul, 02841 Korea; 3 School of Life Sciences, GIST Research Institute, Gwangju Institute of Science and Technology, Gwangju, 61005 Korea

## Abstract

Tissue clearing enables us to observe thick tissue at a single cell resolution by reducing light scattering and refractive index matching. However, imaging of a large volume of tissue for 3D reconstruction requires a great deal of time, cost, and efforts. Few methods have been developed to transcend these limitations by mechanical compression or isotropic tissue shrinkage. Tissue shrinkage significantly lessens the imaging burden; however, there is an inevitable trade-off with image resolution. Here, we have developed the “BrainFilm” technique to compress cleared tissue at Z-axis by dehydration, without alteration of the XY-axis. The Z-axis compression was approximately 90%, and resulted in substantial reduction in image acquisition time and data size. The BrainFilm technique was successfully used to trace and characterize the morphology of thick biocytin-labelled neurons following electrophysiological recording and trace the GFP-labelled long nerve projections in irregular tissues such as the limb of mouse embryo. Thus, BrainFilm is a versatile tool that can be applied in diverse studies of 3D tissues in which spatial information of the Z-axis is dispensable.

## Introduction

Volume imaging using tissue clearing techniques has provided revolutionized tools for the study of 3D tissue architecture at cellular resolution^1–3^. Conventionally, 3D information was obtained by processing of a series of 2D images from physically or optically sectioned single plane images. Compared to physical sectioning which requires extra efforts for serial sectioning and post-alignment of acquired images, optical sectioning with confocal or light-sheet microscopy after tissue clearing is a rapid and efficient alternative method^4^. Tissue clearing uses combinations of lipid extraction and adjustment of refractive index (RI) with surrounding media. Lipid is a major factor in light scattering and can be removed by organic solvents or detergents. More importantly, tissues are mixture of macromolecules with different RI, and the adjustment of tissue and light path with appropriate RI matching solutions greatly affects tissue transparency.

The use of hydrogel polymer for tissue clearing in CLARITY or related protocols has greatly improved the efficiency and reproducibility of tissue clearing techniques^5–7^. Specifically, hydrogel polymer provides tissues with physical strength after lipid removal, allowing isotropic maintenance of tissue size. Furthermore, hydrogel-based clearing techniques can be combined with electrophoresis to expedite the process. Conversely, tissue clearing techniques based on organic solvents, such as BABB, 3DISCO, and iDISCO, have a tendency to shrink samples by dehydration^8–10^. Tissue shrinkage may cause distortion of the sample structure and reduce spatial resolution/accuracy of the images, thus the application of this technique is limited to non-quantitative morphometric analyses^11–13^. However, mechanical compression has been proposed to increase the depth acquisition from a cleared embryo^14^, which also shortens the duration of imaging 3D axonal projections. To further reduce the burden of data collection, a version of iDISCO, uDISCO, has recently been developed to achieve isotropic shrinkage (30–55%) of the sample^15^. Imaging large volumes requires substantially long image acquisition and processing times and high computing power, and isotropic tissue shrinkage can reduce these efforts to obtain large volume information. However, this technique has a trade off with isotropic image resolution.

In this study, we introduce a novel method to reduce the volume of image acquisitions applicable to polymer-based tissue clearing techniques. Acrylamide polymers are highly hydrophilic and absorb a large proportion of water. Therefore, simple drying of the acrylamide gel has long been used in biochemical labs to reduce volume. The unique feature of our tissue compression technique, which we have named “BrainFilm”, is a selective compression of 3D samples in only Z-axis by dehydration. Thus, our BrainFilm technique can be applied to trace single neuronal profiles or axonal projections in a large volume of tissue, and can be applied in various studies such as transgenic animal phenotyping.

## Results

### Overview of the BrainFilm technique

**Figure 1:**
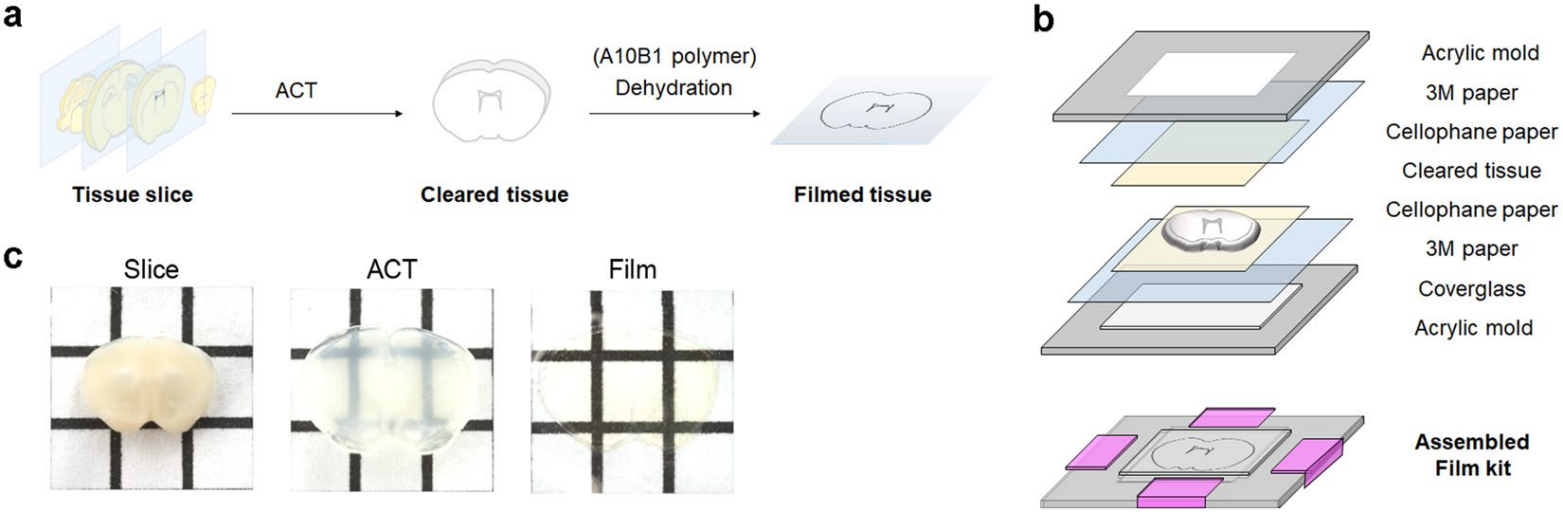
Schematic diagram of the BrainFilm technique. (**a**) Schematic diagram of the BrainFilm process. Fixed tissue slices go through an SDS-based ACT clearing process. The cleared tissue is placed in a BrainFilm kit and placed in a drying oven for dehydration to compressed tissue Film. For tissue thicker than 1 mm, a cleared tissue is incubated in A10B1 solution and solidified with 10% APS and TEMED to provide a supporting structure for tissue during the rocess. (**b**) Film kit contains a set of acrylic mould, coverglass, 3 M paper, and a cleared tissue between cellophane papers. These materials are stacked up as shown above. (**c**) A cleared tissue is dried and compressed in the assembled Film kit within few hours in a dying oven. BrainFilm-processed tissue is then placed on a coverglass for imaging.

Brain slices were fixed, incubated in A4B0 solution, polymerized, and cleared using a routine SDS-based ACT protocol^5^. Cleared brain slices were sandwiched within a compressing tool that we have named “BrainFilm kit.” This kit is composed of an acrylic mould and other easily accessible materials including cellophane paper, 3M paper, and coverslip (Fig. 1b). The assembled BrainFilm kit was placed in a drying oven for dehydration. Within a few hours, a compressed tissue Film is obtained tissue morphology is preserved.

**Figure 2:**
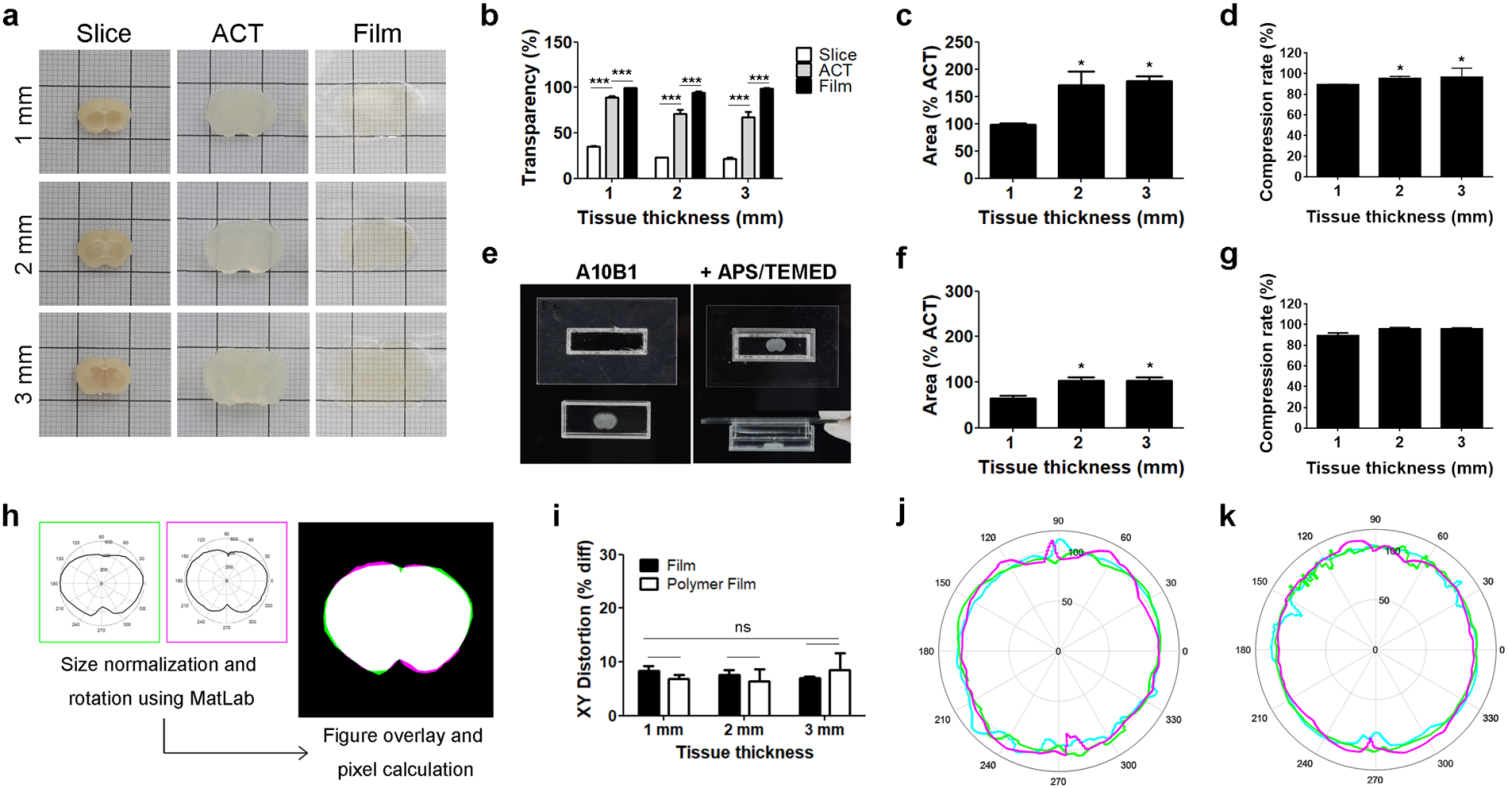
Analysis of tissue transparency, size, and thickness of BrainFilm. (**a**) Images of 1, 2, and 3 mm-thick brain slices after polymerization, ACT, and Film. Each tissue was placed on a 1 cm grid. (**b**) Transparency was normalized with a polymerized tissue sample (n = 10; ***p < 0.001). (**c**) Size expansion was calculated by measuring the area within tissue boundary, and values were normalized with the area of cleared tissues before Film process (n = 10; *p < 0.05). (**d**) Compression rate was measured by scanning the tissue stained with Hoechest33343, and the thickness of BrainFilm sample was normalized by initial tissue thickness (n = 14; *p < 0.05). (**e**) Photos of polymer casting before/after the polymer solidification. (**f,g**) The A10B1 polymer diminished the size expansion of 2 and 3 mm-thick slices during the BrainFilm process (n = 5; *p < 0.05), without changing the compression rate (p = n.s.). (**h**) Stages of the distortion analysis program. Binarized images of tissue before and after BrainFilm process were normalized by initial area and rotated, shown as two images placed in a polar plot. Two images were overlaid, and unmatched pixels were counted using MatLab program. (**i**) Distortion rates of BrainFilm-processed tissues showed no difference due to tissue thickness or polymer existence (n = 3; Two-way ANOVA F = 6.12, p = n.s.). (**j,k**) Representative polar plots of brain slices with or without polymer casting. Different line colors indicate the data from the original thickness of the samples (1 mm, pink; 2 mm, green; 3 mm, blue).

After BrainFilm processing, the transparency of brain slices was substantially increased reaching 100% transparency, regardless of sample thickness within a 1–3 mm range (Fig. 2a,b). We reasoned that dehydrating the tissue reduces the RI differences between polyacrylamide and water in the sample, resulting in increased homogeneity of the materials, reduced light scattering, and increased the transparency. By drying, we achieved approximately 90% compression rate in regardless of tissue thickness (Fig. 2d). However, substantial lateral expansion at the X-Y plane was found, especially in thicker slices (Fig. 2c). Thus, to prevent lateral expansion during the BrainFilm process, ACT-processed brain slices were immerged in polymer to support a large volume of tissue during dehydration and compression (Fig. 2e). With the aid of acrylamide polymer, whose stiffness is similar to intact brain tissues, 2 and 3 mm-thick tissue slices maintained the original XY area after the BrainFilm process, whereas shrinkage was observed in 1 mm slices (Fig. 2f). Thus, the concentration of acrylamide supporting materials should be empirically adjusted to obtain the best condition for the maintenance of size with compression of slice thickness. Regardless of polymer, the BrainFilm-processed samples with polymer maintained high transparency and exhibited 90% reduction in thickness (Fig. 2b,g). Collectively, BrainFilm can be executed with or without supporting polymer casting to achieve the maintenance of tissue size in 2D compared to 90% reduction of Z-axis.

Next, we analyzed whether the BrainFilm produces XY-axis distortion during tissue compression. Binarized images before and after BrainFilm process were automatically normalized by initial area. Using MatLab program, two images were overlayed, and unmatched pixels were labeled and counted to obtain a distortion rate in XY-axis (Fig. 2h). The average distortion was 8.29% in 1 mm-thick tissues, and the distortion rates remained less than 10% in 2 and 3 mm-thick slices (7.60% and 7.06%, respectively) (Fig. 2i). In the presence of A10B0 polymer, similar distortion rates were obtained regardless of tissue thickness (6.80%, 6.45% and 8.44%, respectively). Polar coordinate plots revealed that distortion primarily occurred in dorsal and ventral regions of the brain slices (Fig. 2j,k), potentially due to the complex curvature in these areas.

### Application of BrainFilm technique to other organs

**Figure 3:**
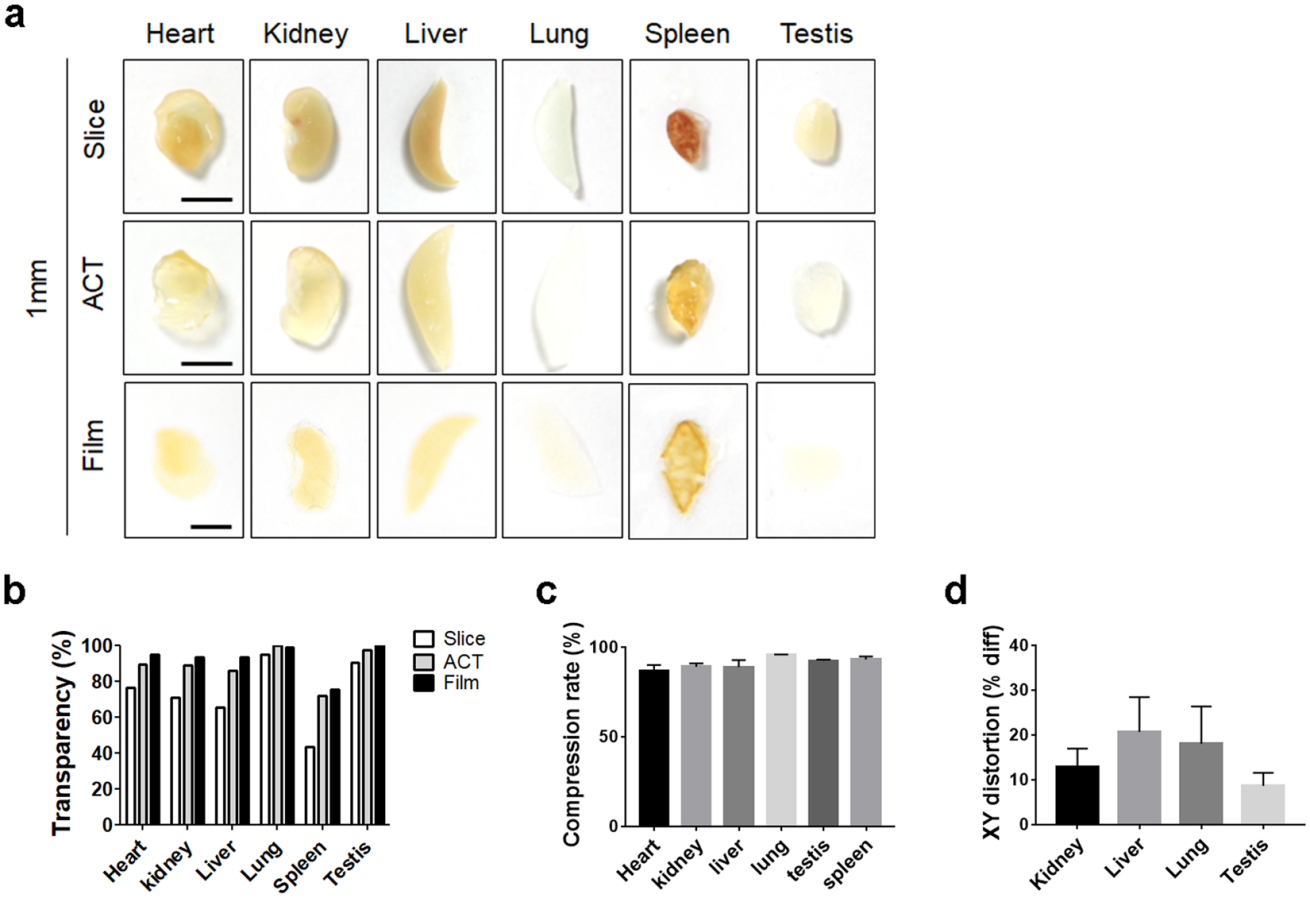
BrainFilm with organ samples. (**a**) Images of 1 mm-thick organ slices after polymerization, ACT, and Film. (**b**) Transparency was normalized with a polymerized tissue sample. (**c**) Thickness was measured by scanning the tissue stained with Hoechest33343, and values were normalized with initial tissue thickness. (**d**) Distortion rates of BrainFilm-processed organ slices (p = n.s.).

In addition to brain slices, various mouse organs can be compressed using the BrainFilm procedure (Fig. 3a). All 1 mm-thick organ slices reached high transparency using this technique, except for spleen tissue due to its pigmentation (Fig. 3b). By complete dehydration, we obtained a compression rate of 87–95%, and XY-distortion rates were less than 20% (Fig. 3c,d). Collectively, these data indicate that Film technique can be used for the examination of various organs, although empirical optimization of the procedure might be required for each organ.

### Single cell tracing using BrainFilm

**Figure 4:**
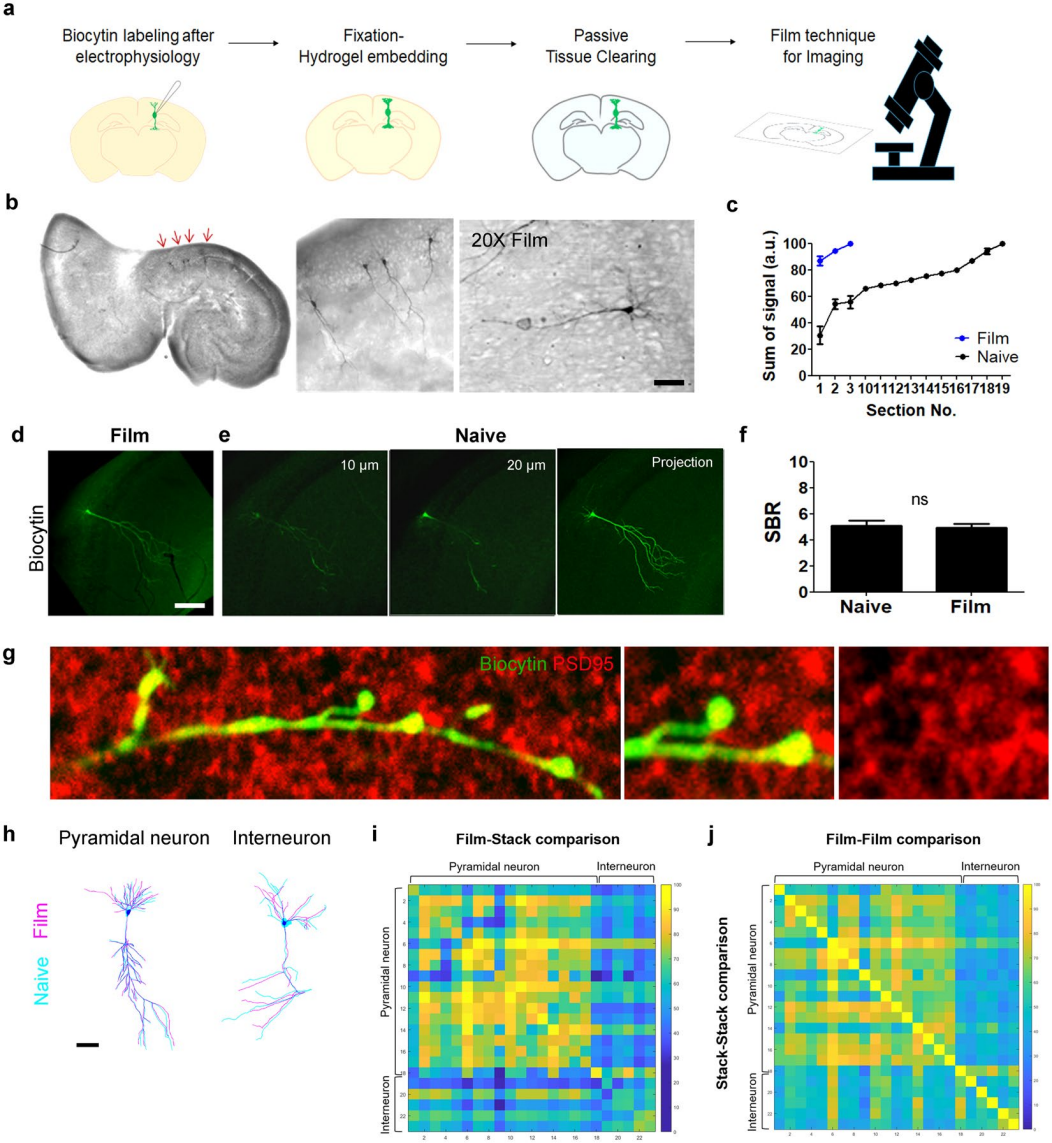
Single cell tracing after BrainFilm technique. (**a**) Schematic diagram of biocytin-labelled cell tracing using the BrainFilm technique. (**b**) DAB stained pyramidal neurons were imaged with an optical microscope. (**c**) Each image of a single neuron was collected in 5 µm intervals, and signal intensity was measured for each image. The maximum intensity of a single neuron was reached within 15 µm. (**d**) Images of the biocytin-labeled neuron after the BrainFilm process. A single image of a labelled cell in BrainFilm-processed tissue provided an image equivalent to the projection image on the right. (**e**) Images of the same neuron at indicated interval and a projection image obtained by a confocal microscope. Biocytin-labelled neurons were immunostained with anti-streptavidin-488. (**f**) Signal-to-background ratio (SBR) show no difference before and after the BrainFilm process (p = n.s.). (**g**) High magnification images of distal neurites and synaptic boutons before and after the BrainFilm process. (**h**) Images of YFP + neurons in the cortex of Thy1-YFP mice followed by the BrainFilm process and additional image processing. (**i**) Heatmap of matching rates between reconstructed neurons obtained from BrainFilm and stack-projection (18 pyramidal neurons, 5 interneurons). (**j**) Heatmap of matching rates between reconstructed neurons derived from BrainFilm or stack-projection.

Biocytin labelling is widely used after electrophysiological recordings of neuronal activity to identify morphological characteristics of the recoded neurons^16^. Since 3D information is dispensable for this application, we tested the feasibility of the BrainFilm technique for obtaining the morphology of biocytin-labelled neurons in 350 µm-thick brain slices (Fig. 4a). Biocytin-labelled neurons can be visualized by colorimetric staining, and entire neuronal morphology was easily captured using a conventional bright field optical microscope (Fig. 4b). For a more precise comparison of naïve and BrainFilm samples, we imaged biocytin-labelled neurons before and after the BrainFilm process using a confocal microscope at 5 µm intervals (Fig. 4d). A projection image was acquired to show the morphology of a single neuron. With the same process as above, we obtained projection images of the BrainFilm-processed tissue. To assess the morphological information of a single neuron from both methods, we rearranged the projection images by eliminating every 5 µm image serially and summed the fluorescent intensity of each projection images (Fig. 4c). To achieve maximum intensity, summation of 3 images from the BrainFilm-processed tissue was equivalent to the summation of 20 images from naive tissue. A single image of BrainFilm is capable of capturing 90% of projected neuronal morphology. Comparing the thickness of two samples demonstrated that BrainFilm-processed image produced a 13-fold greater compression of image information. Additionally, the signal-to-background ratio (SBR) did not significantly change after the BrainFilm process (4.93 ± 0.32 vs. 5.09 ± 0.40, p = n.s.) (Fig. 4f), providing an accurate image at a given focal depth. Moreover, a higher resolution image of a biocytin-positive pyramidal cell exhibited synaptic boutons on distal neurites (Fig. 4g). Taken together, our data demonstrates that the BrainFilm technique can be used to detect clearly visible signals with fewer images, due to the concise image processing provided by compressed tissues, which enables efficient visualization of individual neurons in a large volume of tissue.

### Cell morphology analysis using BrainFilm

To assess compression induced distortion of neuronal morphology, we compared the morphology of 17 different pyramidal cells and 5 interneurons in projection images using modified NeuroGPS-tree method^17^ (Fig. 4h, Supplementary Fig. 1). The matching rate is defined as the ratio of the number of true positive points to the number of any given points on the manually reconstructed skeleton. A heatmap of matching rates between reconstructed neurons from BrainFilm and stack-projection indicated that the matching rates of pyramidal neurons were higher than that of interneurons (83.09 ± 5.86 vs. 66.69 ± 3.52, p < 0.0001) (Fig. 4i). We also compared the morphology of reconstructed neurons within BrainFilm or stack-projection group. A heatmap of matching rates from each group exhibited a similar pattern that distinguished pyramidal neurons from interneurons (Fig. 4j). The matching rates of pyramidal neurons in BrainFilm-processed images were comparable to those from stacked images (63.23 ± 0.80 vs. 63.14 ± 0.87, p = n.s.). However, the matching rates of interneurons in BrainFilm-processed images were decreased due to distinct morphological differences (48.37 ± 2.18 vs. 52.17 ± 1.84, p = n.s.). In addition, manual reconstruction of cell morphology in a single BrainFilm image, captured more details in basal dendrites compared to the complete cell morphology obtained from each slice images (Supplementary Fig. 2). Our data shows that the BrainFilm technique provides an accurate and precise analysis of single cell morphology in the brain.

### Applications to GFP-labelled axonal tracing

**Figure 5:**
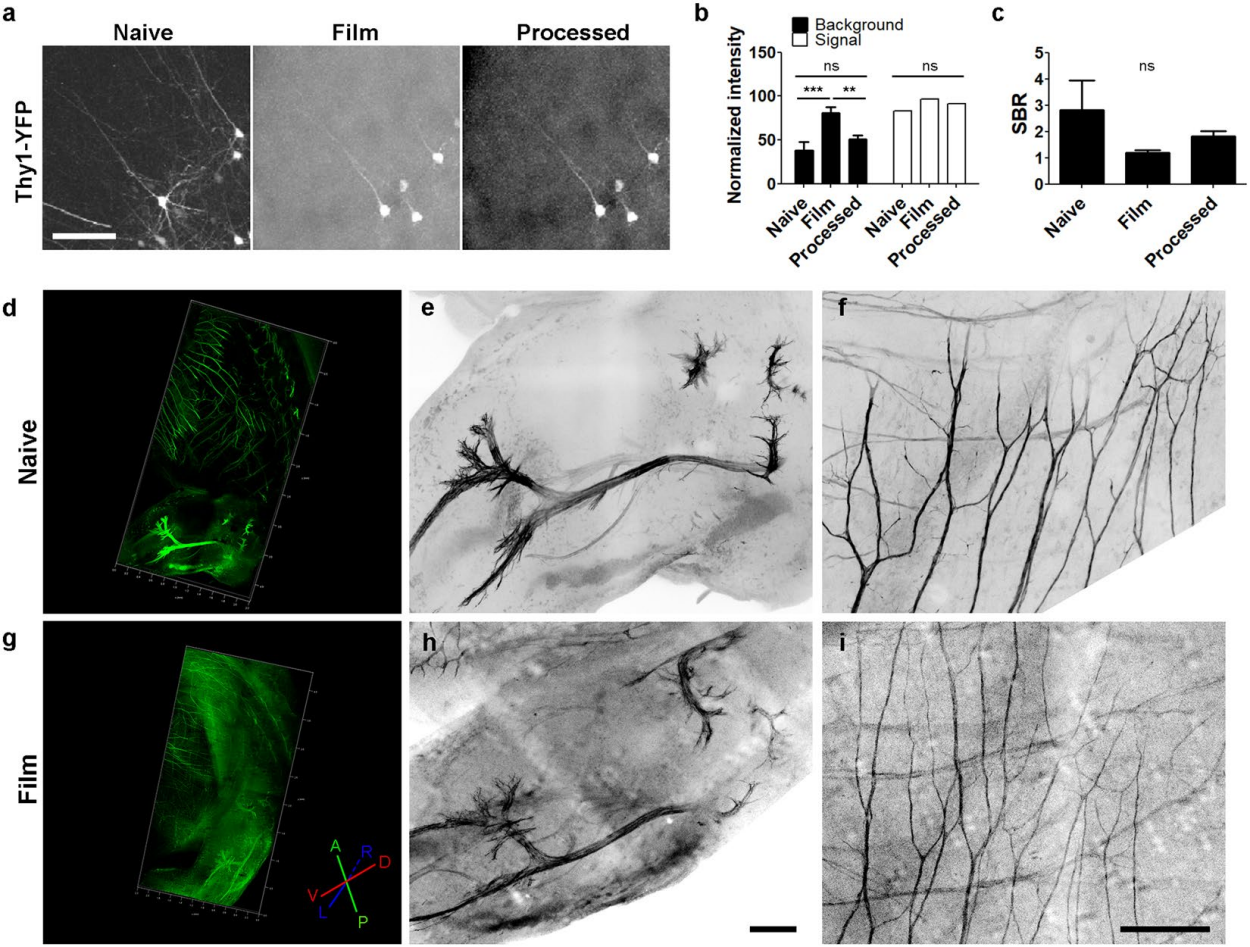
Observation of nerve connections in Thy1-YFP brain slice and the hindlimb and lumbosacral regions of E13.5 mouse embryo using BrainFilm technique. (**a**) Images of YFP + neurons in the cortex of Thy1-YFP mice followed by the BrainFilm process and additional image processing. (**b**) Normalized intensity obtained from YFP + neurites and background (n = 3; Two-way ANOVA F = 4.40, p = 0.0369; **p < 0.01, ***p < 0.001). (**c**) Signal-to-background ratio (SBR) was different before and after the BrainFilm process (n = 3). (**d,g**) 3D images of the same HB9-GFP mouse embryo before and after the BrainFilm process. (**e,f**) Enlarged images of the lumbosacral nerve plexus. The path of the nerve bundle is well presented in the BrainFilm-processed embryo. (**f,i**) Enlarge images of cutaneous maximus innervation perpendicular to intercostal nerves. Scale = 500 µm.

Next, we applied the BrainFilm technique to 1 mm Thy1-YFP mouse brain slices. The image in XZ and YZ plane of a 3D figure indicates isotropic compression in the Z-axis. Fluorescence signals were well maintained after the BrainFilm process. Due to the high contents of YFP signals, background-like signals were intensified by compression of thick tissue, resulting in a reduced SBR used to identify individual cell morphology and image post-adjustment was required to enhance the SBR (Fig. 5a–c). Next, we traced GFP-labelled peripheral projections in E13.5 HB9-GFP embryo (Fig. 5d,e). In this application, the imaging area was large, and the shape and thickness of the sample were irregular. Acrylamide polymer was applied to support the irregular tissue, resulting in sufficiently even compression and dehydration in the Z-axis. However, the hindlimb and lumbosacral region of E13.5 GFP mouse embryo were successfully imaged from the BrainFilm-processed specimen and was sufficient to identify the lumbosacral plexus, cutaneous maximus and intercostal nerves at thoracic levels.

Additionally, we evaluated whether Film technique can be applicable to the large specimen with irregular 3D morphology such as cerebellum (Supplementary Fig. 3). We discovered that the whole mouse cerebellum was successfully compressed, but relatively large variation in compression rate was seen especially at the edge of the specimen, presumably owing to the irregular morphology of this area (Supplementary Fig. 3d,e). In addition, distortion rate at XY-axis was also large comparing to the data from 1–3 mm tissue slices (Supplementary Fig. 3c vs. Fig. 1d,g). Despite the limitation, the time saving aspect of BrainFilm is most beneficial in case of imaging a large volume. Imaging the cleared mouse embryo required 12 hours with 2. 41 µm intervals and 3 × 4 tile size, whereas the imaging duration was significantly reduced to 2 hours after the BrainFilm process using the same interval and tile size. Accordingly, data size was substantially reduced when imaging the BrainFilm-processed embryo.

## Discussion

The development of tissue clearing techniques has allowed for imaging tissue depth with high resolution. Simultaneously, demands for data acquisition and post-processing have increased to reconstruct whole organ structure with detailed cellular compositions. To reduce imaging duration, the Deisseroth group integrated light-sheet microscopy to accelerate data acquisition from cleared samples^18,19^. Although high speed light-sheet microscopy maintains the quality and resolution of image, the size of imaging data is exponentially increased when examining the whole rodent brain. Thus, physically down-sizing tissue volume would be also lessen the imaging burden. The property of isotropic shrinkage in specific organic solvents by strong dehydration has successfully achieved and been used to reduce tissue volume, resulting in accelerated data acquisition and post processing.

CLARITY or ACT are alternative tissue clearing techniques that use acrylamide polymer infusion to produce a tissue-hydrogel complex^5,6^. Polyacrylamide is widely used to make gels for biochemical separation of proteins, and drying the gel for further analysis or storage is an old trick. Thus, we designed the BrainFilm technique to dehydrate and compress hydrogel-based cleared tissue sample to generate a projection-like images, and significantly reduce imaging burden. Our technique enables even compression of tissue samples on the Z-axis by approximately 90%, without changing the size or distorting the shape of the XY-axis. Thus, a substantial reduction in the Z-axis reduces the time and effort needed for image acquisition and data processing. Alternatively, maintaining size in the XY-axis allows for precise and high-resolution morphometry of neurons in thick brain slices. The Z-axis compression rate of 90% provides a projection image of whole cell in a single shot, while often obtaining high-resolution images in which nearly the entire dendritic morphology of single neurons can be seen even without confocal microscopy. Due to the nature of physical compression, an inevitable reduction in the SBR, especially in tissue samples with abundant expression of fluorescent proteins. Thus, this technique is especially suitable for 1–2 mm sliced samples, and additional post-image processing algorithms should improve image quality. Since the entire mouse brain can be converted to 12–15 slices of 1 mm sections, and semi-automatically processed with BrainFilm, we propose that our technique is also suitable for large-scale systematic analysis of brain phenotyping.

Although BrainFilm is suitable for the sliced brain tissues, we could successfully expand this technique to other organs, and irregular samples such as mouse embryo, indicating that this novel approach is not limited for use in brain slice imaging and can also be used for any application in which the benefit of image compression is greater than the reduction of Z-axis resolution.

## Materials and Methods

### Animals

For electrophysiological studies, Sprague-Dawley rats (post-natal days 14–28) were decapitated under deep isoflurane-induced anaesthesia in accordance with the Institutional Animal Care and Use Committee of Korea University (KUIACUC-20150520-1). For organ clearing, adult C57BL/6 mice (DAEHAN Biolink, Inc.) and Thy1-YFP mice (B6.Cg-Tg(Thy1-YFP)16Jrs/J) were anesthetized with urethane and transcardially perfused with 0.9% saline followed by 4% paraformaldehyde. For mouse embryo, E13.5 HB9-GFP were sacrificed and processed in GIST^20^. All animal maintenance and experimental procedures were approved by members of Laboratory Animal Research Center in Korea University College of Medicine and the Animal Care and Ethics Committee of the GIST.

### Slice preparation and biocytin infusion

Brains were removed from rats and quickly submerged in ice-cold artificial cerebro-spinal fluid (aCSF) containing 126 mM NaCl, 3 mM KCl, 1.25 mM NaH_2_PO_4_, 2 mM MgSO_4_, 2 mM CaCl_2_, 25 mM NaHCO_3_ and 10 mM glucose (pH 7.2–7.4) which was oxygenated with carbogen gas (95% O_2_, 5% CO_2_). A vibratome (VT1000s, Leica) was used to cut 350 μm-thick horizontal hippocampal slices. Slices were incubated in oxygenated aCSF for at least 1 hour at room temperature before being transferred to a recording chamber under the microscope (BX51, Olympus) superfused with oxygenated aCSF at room temperature. Whole-cell patch-clamp recordings were made from hippocampal CA1 pyramidal cells and interneurons with MultiClamp700B amplified (Molecular Device) in current-clamp mode under the visual guidance of infrared differential interference video microscopy. Patch electrodes were pulled from borosilicate glass capillaries (tip resistance: 4–8 MΩ) and filled with intracellular solution containing 110 mM K-gluconate, 40 mM HEPES, 4 mM NaCl, 4 mM ATP-Mg, 0.3 mM GTP and 4 mg/ml biocytin (pH 7.2–7.3, 270–300 mOsm/L). Large depolarizing currents (100 ms, 1 nA, 1 Hz) were applied for at least 40 min to expedite the diffusion of biocytin throughout the neurites of the patched neuron. Brain slices were left in the recording chamber for an additional 10 min after recording to wash out excess biocytin from the extracellular space.

### Tissue clearing

For tissue clearing, we used 0.3, 1, 2, 3, and 5 mm brain slices and 1 mm-thick organ tissues. After electrophysiological recording, 0.3 mm brain slices were used to acquire biocytin-filled neuron images. Brain slices with 0.3 mm were fixed with 4% PFA for 1 h at room temperature, and others were fixed overnight at 4 °C. Samples were washed 3 times with 0.1 × PBS, incubated with polymerization buffer (A4B0; 4% acrylamide in 0.1 × PBS supplemented with 0.25% the photoinitiator 2,2′-azobis[2-(2-imidazolin-2-yl)propane] dihydrochloride (Wako Pure Chemical, Osaka, Japan) overnight at 4 °C. Samplers were degassed for 5 min and polymerized for 3 h at 37 °C, and samples were washed in 0.1 × PBS to remove excess hydrogel. Samples were then placed in 8% SDS for 2 h at room temperature while shaking. For brain slices thicker than 1 mm, we used the ACT protocol previously described^5^. Briefly, tissue slices were post-fixed in 4% PFA overnight at 4 °C and incubated in A4B0 solution. After polymerization, samples were placed on a tissue container in the ETC chamber and processed with following conditions: 1.5 mA, 37 °C, time depending on sample thickness (1 h for 1 mm brain slice and a 1 h increase for each 1 mm thickness, 3~6 h for 1 mm organ slices). After tissue clearing, all samples were washed in 0.1 × PBS overnight at room temperature while shaking to remove SDS.

### Immunostaining

For immunostaining, tissue samples were incubated with primary antibody against PSD95 diluted in 6% BSA and 0.2% Triton-X100 in 0.1 × PBS for 2 days at 37 °C on a shaker. Samples were rinsed 3 times with 0.1 × PBS and incubated with the appropriate secondary antibodies for 2 days at 37 °C on a shaker. Biocytin-labeled pyramidal neurons were stained with 488 conjugated streptavidin. After washing, tissue slices were incubated in CUBIC-mount for 1 h for imaging. Stained tissue samples were then washed in 0.1 × PBS prior to BrainFilm procedure.

### BrainFilm procedure

We designed an acrylic kit to evenly compress cleared tissue slices and convert them to BrainFilm. On top of an acrylic mould, a coverglass and 3 M paper were placed in consecutive order. Cellophane paper (Bio-Rad) was placed on 3 M paper, and distilled water was applied on top to flatten the cellophane paper. Cleared tissue slices or tissue with polymer were placed on the cellophane paper. The sample was sealed with cellophane paper, and distilled water was applied to remove bubbles. Another sheet of 3 M paper was placed on top of sample, followed by an acrylic mould. The BrainFilm kit was assembled with clips on every side and placed in an oven at 55 °C for 1 hr to a day, depending on tissue thickness. For tissue slices thicker than 2 mm, samples were incubated in 10% acrylamide and 0.1% bis-acrylamide in 0.1 × PBS (A10B1) overnight at room temperature. The new A10B1 solution with 10% APS and 0.05% TEMED was poured over the tissue, and the solution was solidified to act as a support fixture for surrounding tissue. The BrainFilm technique was also applied to the tissue in the gel. After the sample was completely dehydrated, the thinned sample covered with cellophane paper was kept between two coverglass slips to preserve its flat surface before observation.

### Imaging

Cleared tissues were incubated in CUBIC-mount solution for 1 h until samples became transparent. Gross images were captured using a digital camera (Cannon, Tokyo, Japan) before and after the BrainFilm process. Images of fluorescently labelled cells in cleared tissue were acquired using a TCS SP8 confocal laser scanning microscope (Leica, Wetzlar, Germany) with an X10 lens (N.D. 0.4; W.D. 2.2 mm) and X25 lens (N.D. 0.95; W.D. 2.4 mm). Z-stack images were converted to a 3D images using the Leica LAS X program, and projection images were processed with Adobe Photoshop CS6. To calculate the signal-to-noise ratio for each image, intensity profiles of single images were obtained with ImageJ.

### Distortion analysis

Boundaries were first drawn onto images of cleared tissue, and marked regions were colored white using ImageJ. First, the area of the larger image was matched to that of the smaller image. To compare the difference in overall shape between the two images, unmatched pixels were counted after images were centralized and overlapped. Local distortions were also calculated by comparing the radial distance of each angle bin (1 degree) in polar coordinates. All data were collected using MatLab R2017a program.

### Mismatch analysis

The morphological similarity of the same neuron between a z-stack image and film was analysed using a custom-made script in Python. The analytical procedure was adapted from the NeuroGPS-tree method^17^. Briefly, neuron morphology was manually traced from the stack and the film image to reconstruct a skeleton with a rounded cell body. To find the matched angle between the two images, we first located the cell body in each image by applying a morphological filter to locate its centroid. While rotating one of the images on the matched centroid, we searched the nearest pixel on the stack image for all of the points on the film image. We found the angle with minimum total distance and applied a geometric transform to the film image. Next, each point on the film image was determined as true positive if the distance to the nearest point on the stack image was less than the threshold (15 um). Matching rate was calculated by dividing the number of true positive points by the number of foreground pixels in the film image and that in the stack image.

### Statistical analysis

Data were analyzed with Graphpad Prism software, and results were presented as mean ± SEM. The Shapiro-Wilk test for normality was performed prior to following statistical analyses. Comparisons were performed using unpaired two-tailed Student’s t-test, one-way ANOVA followed by Tukey’s *post hoc* test or two-way ANOVA followed by Bonferroni post-test when appropriate. A *p*-value of < 0.05 was considered statistically significant.

## Electronic supplementary material

Supplementary Information

Supplementary Video 1.
