# Supplementary material for: BrainFilm, a novel technique for physical compression of 3D brain slices for efficient image acquisition and post-processing
Source: Sci Rep. 2018 Jun 4;8:8531. doi: 10.1038/s41598-018-26776-9 (PMC5986777; doi:10.1038/s41598-018-26776-9)
Supplement: Supplementary file 1 — Supplementary Information [file 41598_2018_26776_MOESM1_ESM.pdf]

Scientific Reports

Supplementary Information

BrainFilm, a novel technique for physical compression of 3D brain slices for efficient image acquisition and post-processing

Joo Yeon Kim[1,4], Hyun Jung Kim[1,4], Min Jee Jang[1], June Hoan Kim[1], Ju-Hyun Lee[1], Eunsoo Lee[1], Kyerl Park[2], Hyuncheol Kim[2], Jaedong Lee[2], Jeehyun Kwag[2], Namhee Kim[3], Mi-Ryoung Song[3], Hyun Kim[1], Woong Sun[1*]

[1]Department of Anatomy and Division of Brain Korea 21 Plus Biomedical Science, College of Medicine, Korea University, Seoul, 02841, Korea
[2]Department of Brain and Cognitive Engineering, Korea University, Seoul, 02841, Korea
[3]School of Life Sciences, GIST Research Institute, Gwangju Institute of Science and Technology, Gwangju, 61005, Korea
[4]Two authors contributed equally to the work.

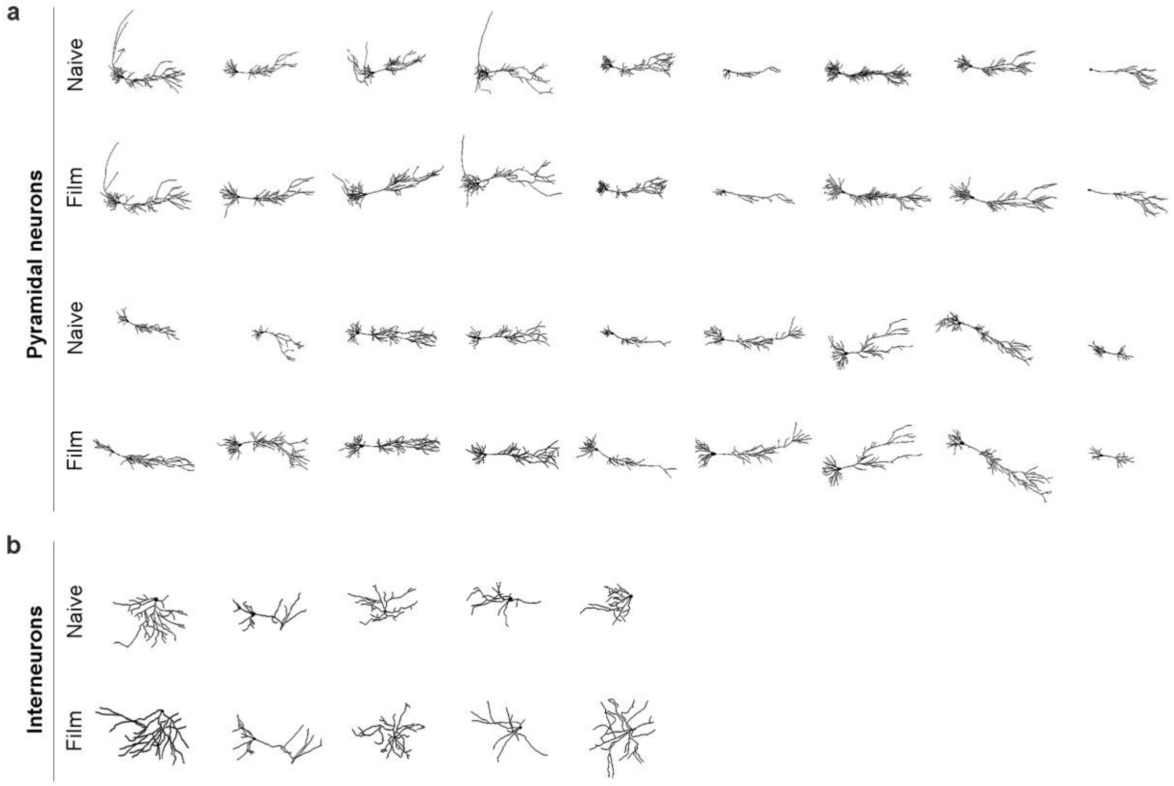

Supplementary Figure 1. Comparison of reconstructed **(a)** pyramidal neurons and **(b)** interneurons from BrainFilm (lower) and stack-projection (upper).

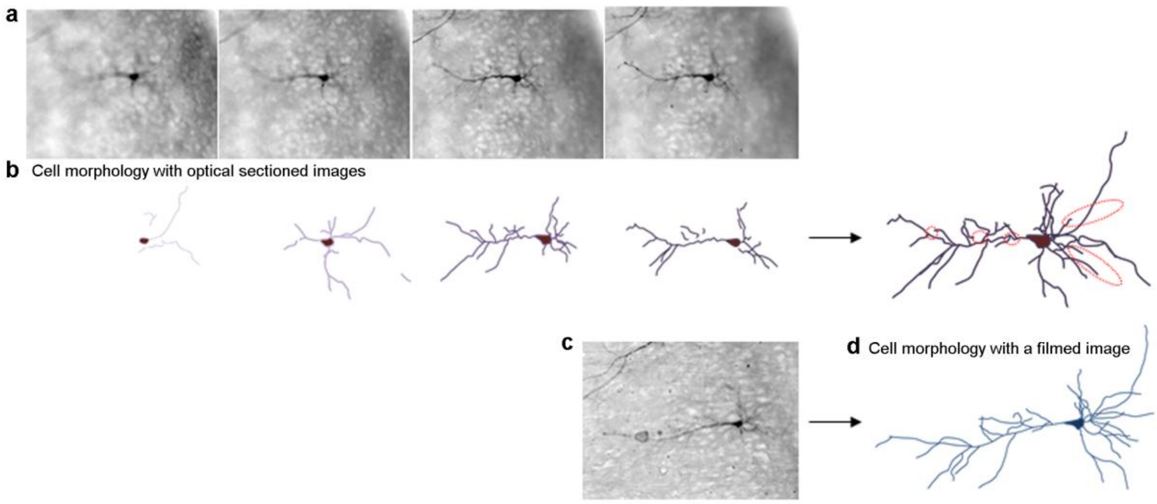

Supplementary Figure 2. Manual reconstruction of a single cell morphology in **(a.b)** stack images and **(c,d)** BrainFilm images. The red circles indicate loss of neuronal arborization information acquired from stack images.

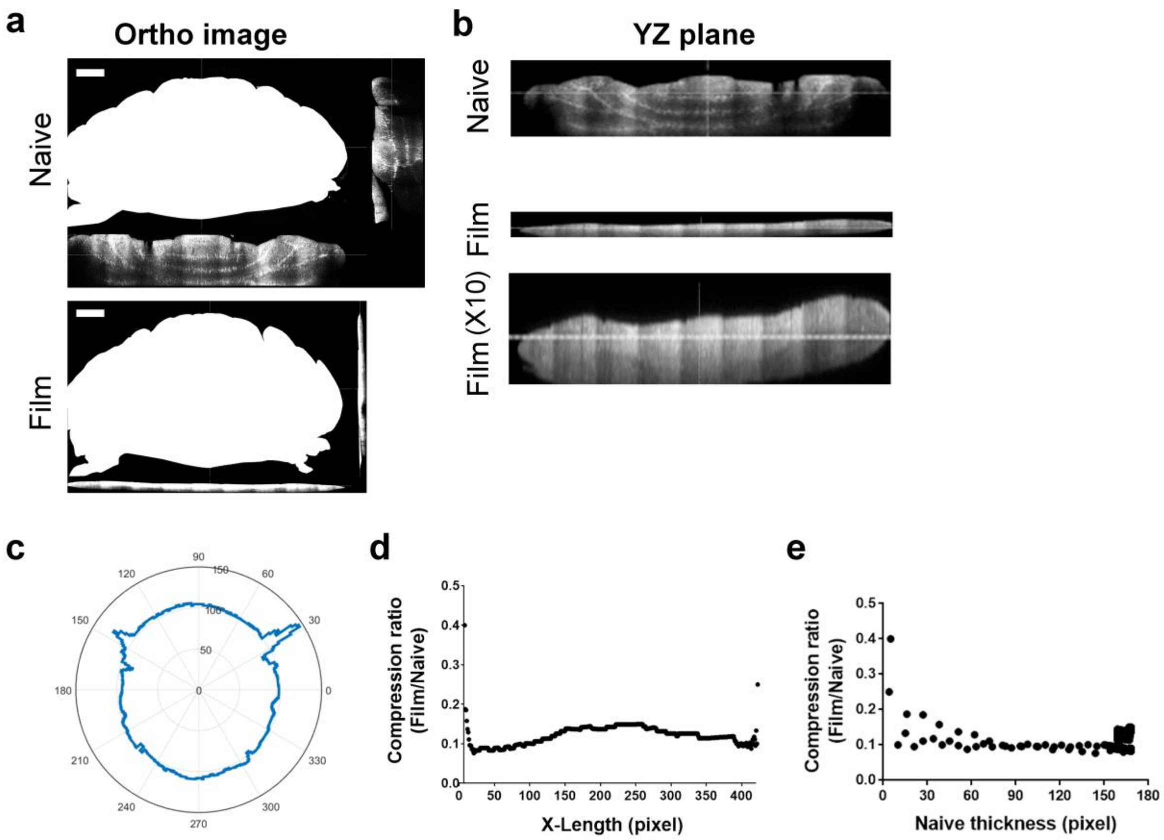

Supplementary Figure 3. Distortion analysis of the Z-axis using Thy1-YFP mouse cerebellum. **(a)** Ortho images and **(b)** XZ plane images before (top) and after (middle) the BrainFilm process (scale = 1 mm). Bottom panel shows the digital expansion (10x) of Filmed XZ image to match the original thickness. **(c)** Polar plot represents slight distortion in XY-axis. **(d)** Compression ratio along the X-length of entire cerebellum and **(e)** the Z-axis compression ratio depending on the original naïve sample thickness of each X-length point was performed by the distortion analysis using MatLab program.
